# The quick are the dead: pheasants that are slow to reverse a learned association survive for longer in the wild

**DOI:** 10.1098/rstb.2017.0297

**Published:** 2018-08-13

**Authors:** Joah R. Madden, Ellis J. G. Langley, Mark A. Whiteside, Christine E. Beardsworth, Jayden O. van Horik

**Affiliations:** Centre for Research in Animal Behaviour, Psychology, University of Exeter, Exeter EX4 4QG, UK

**Keywords:** pheasant, *Phasianus colchicus*, reversal learning, associative learning, survival, fitness

## Abstract

Cognitive abilities probably evolve through natural selection if they provide individuals with fitness benefits. A growing number of studies demonstrate a positive relationship between performance in psychometric tasks and (proxy) measures of fitness. We assayed the performance of 154 common pheasant (*Phasianus colchicus*) chicks on tests of acquisition and reversal learning, using a different set of chicks and different set of cue types (spatial location and colour) in each of two years and then followed their fates after release into the wild. Across all birds, individuals that were slow to reverse previously learned associations were more likely to survive to four months old. For heavy birds, individuals that rapidly acquired an association had improved survival to four months, whereas for light birds, slow acquirers were more likely to be alive. Slow reversers also exhibited less exploratory behaviour in assays when five weeks old. Fast acquirers visited more artificial feeders after release. In contrast to most other studies, we showed that apparently ‘poor’ cognitive performance (slow reversal speed suggesting low behavioural flexibility) correlates with fitness benefits in at least some circumstances. This correlation suggests a novel mechanism by which continued exaggeration of cognitive abilities may be constrained.

This article is part of the theme issue ‘Causes and consequences of individual differences in cognitive abilities’.

## 1. Introduction

One powerful approach to understand how natural selection may act on cognition is to measure the performance of individuals in a particular cognitive domain, and then explore how their performance correlates with a (proxy) fitness measure [1,2]. This is achieved by deploying explicit psychometric tasks targeting specific, defined cognitive processes [3,4]. Because fitness itself is hard to measure [5], researchers tend to use proxies that are presumed to correspond to reproductive success and/or survival. This correlational approach has predominantly revealed a positive relationship between an individual's performance in the psychometric task and a (proxy) measure of their fitness. Ants *Lasius niger* that exhibited faster route learning had greater colony-level foraging success [6]. Male African striped mice (*Rhabdomys pumilio*) that escaped quickly from mazes also had increased probability of surviving to the breeding season [7]. Male guppies (*Poecilia reticulata*) that learned mazes quickly were preferred by females [8]. Male bitterling (*Rhodeus ocellatus*) (practising a ‘sneaker’ strategy) that exhibited better maze learning subsequently had higher reproductive success [9]. Male song sparrows (*Melospiza melodia*) that demonstrated better control in a detour-reaching task had a larger song repertoire [10]. Male starlings (*Sturnus vulgaris*) with better spatial learning exhibited longer song bouts [11]. One study of female Australian magpies (*Cracticus tibicen dorsalis*) reported a link between their reproductive success and a general factor summarizing their performance in a battery of four tasks [12]. By contrast, a study of spotted bowerbirds (*Ptilonorhynchus maculatus*) found no relationship between a male's mating success and his performance in a battery of six tasks, either individually or when his performance was summarized by a single component [13]. Only one study has reported a negative relationship: male song sparrows that were fast at spatial learning also had smaller song repertoires [14]. This implies that natural selection generally leads to more exaggerated cognitive performance and associated abilities.

Interpretation of these previous studies is complicated by three factors. First, in all cases except one [12], a single assay has been used for each cognitive process being investigated. Reliance on a single assay risks a misattribution of the mechanisms driving individual performance. For example, learning to discriminate between two colour cues may indicate the specific ability or inherent motivation to prefer one colour over another [3], rather than the more general ability to learn associatively. A more robust method would be to use two (or more) tests that assay the same putative cognitive mechanism but differ in format or cue uses and hence triangulate on the outcome (Volter *et al.* [15]). We considered two ubiquitous cognitive processes and tested each using two different test variants. Associative learning involves learning to associate a stimulus with a reward and may be tested using a binary discrimination. Reversal learning may be measured by the speed at which such a previously learned association can be reversed. Reversal learning is considered to indicate an individual's ability to exert executive, inhibitory control and thus be behaviourally flexible ([16,17] corvids (*Gymnorhinus cyanocephalus, Nucifraga columbiana, Aphelocoma californica*); but see also [18] humans). The processes have been linked to specific behaviours and fitness consequences. Associative learning performance determines adult foraging strategies ([19] sparrow, *Passer domesticus*) and rapid learning speeds enhance individual's foraging or reproductive success ([20,21] grasshopper, *Schistocerca americana*; wasp, *Biosteres arisanus*). Flexibility permits rapid switching between different optimal decisions in changeable environments [22] so that more behaviourally flexible individuals have improved invasion success ([23] Birds) or a better ability to track fluctuating social groups ([24] Primates). The two processes (associative learning and reversal learning) may be closely related to one another. In several other species, speeds of associative learning and reversal learning are negatively related ([25–27] myna, chickadee, scrub-jay (*Aphelocoma coerulescens*), red junglefowl (*Gallus gallus*)). However, this negative relationship is not inevitable ([13,28] bowerbird, robin (*Petroica longipes*)) and indeed may be positive [29] or moderated by another factor e.g. testosterone [25].

Second, previous studies have not attempted to explicitly test how performance in abstract cognitive tasks relates to specific behaviours upon which selection may act. For example, it is not clear how improved inhibitory control as revealed by performance in a detour task may relate to song-learning processes [10], or how the ability to navigate a maze manifests in improved mating success [9]. One possibility is that cognitive performances and natural behaviours are linked by an overarching personality, such that an individual's behaviour in one context (a cognitive task) is linked to their behaviour in another context [30,31]. Alternatively, a cognitive ability has an immediate link to a natural behaviour, independent of personality. For example, performance in maze learning may correspond to the methods by which an individual learns to navigate their environment and recall feeding and refuge locations. By explicitly testing how cognitive abilities relate to broader personality assays, or more specific behaviours likely to relate to fitness outcomes, we can better understand how selection may act on these abilities.

Finally, studies have either had to test wild individuals for whom prior experience, social ranking and/or age is unknown, or they have relied on laboratory systems where the putative fitness consequences are hard to relate to the natural world. Administering controlled psychometric tests to wild animals, in which a large, random and reasonably complete sample of individuals participate over a large number of repeated presentations is problematic [1–4]. One solution is to capture animals from the wild and take them into captivity where they can be tested before release back into the wild. This approach encounters two problems. First, capture may not be random [32], so that the sample tested is not representative of the wild population. Second, individuals may have undergone different prior experiences that could lead to biases or preferences (e.g. for a particular colour) developed in other contexts that skew their performance in tests [3]. Such problems may be overcome by testing captive-reared individuals where prior experiences can be controlled and participation ensured. However, captive animals are not subject to natural selective pressures because predators are excluded and resources are provided in excess, and hence robust and relevant fitness measures are difficult to collect. This may explain why previous studies have used proxy measures of fitness.

We made use of a unique study system, the common pheasant (*Phasianus colchicus*) (hereafter pheasants). In the UK, these birds can be reared in captivity from hatching and subsequently released into the wild (for hunting). This ensures that individuals all experience identical developmental trajectories and prior experiences, all can be tested under controlled conditions and, critically, after release can be subject to natural selective pressures in the wild, where their fates can be monitored. We reared pheasant chicks from hatching to 10 weeks under controlled conditions in 2014 and 2015, and during this time we could subject them to psychometric tests of acquisition and reversal learning [33]. We used two sets of tests of particular processes, specifically the acquisition and reversal of associations between cues and rewards, using two different task paradigms (one discriminating colours and the other discriminating spatial positions on the test apparatus), with one task paradigm used in each year, to improve our confidence that it was the cognitive process that we were measuring rather than simply response to one particular set of cues. Critically, we then released birds into the wild and followed their fates, using survival as an unambiguous indicator of their fitness. Pheasant survival may be affected by year [34,35], sex ([36,37], but see also [38]), mass ([39], but see also [40]) and interactions between them (e.g. [41]). Therefore, we considered these in conjunction with performances in the cognitive tests. Pheasant mortality is typically high, especially in early life when birds are first independent, due to both terrestrial and avian predators [41,42]. This mortality occurs when pheasants disperse from their open-topped release pens and hence encounter novel predators and move away from artificial food provision. Pheasants that leave such safe release sites and fail to learn new foraging locations or refuges from predators are likely to be highly susceptible. We asked whether survival was predicted by a pheasant's early life performance in psychometric tests of learning and reversal, controlling for other non-cognitive factors such as sex and mass. Given the ambiguous relationship previously reported between an individual's speed of acquisition and reversal [13,25–29], we tested how performances in these two tasks were related to each other in pheasants. We then explored two mechanisms by which any such relationships between cognition and fitness may be mediated by their movement and exploration. As pheasants moved further away from their point of release (in a protected and provisioned pen—see below), they would encounter higher densities of predators and lower densities of artificial food supplies, and hence face an increased risk of predation or starvation. First, we tested how an individual's exploratory behaviour in a series of assays under controlled conditions when five weeks old correlated with their cognitive ability. Second, we tested how early life cognitive performance related to adult ranging behaviour after release.

## 2. Methods

### (a) Housing

In each of two years, 200 day–old pheasant chicks were housed in groups of 50 in four replicated enclosures at North Wyke Farm, Devon, UK and reared from one day old between 28 May and 29 July 2014, and 27 May and 29 July 2015. See electronic supplementary material for details of chick origins, housing and rearing practices. The mass of each bird was collected when the birds were 9–10 weeks old and sex confirmed by plumage features prior to release.

### (b) Training and testing

All chicks were habituated to enter the testing arena of their own volition when an enclosure-specific whistle was given. The door was then closed allowing for testing in isolation. Subjects were initially trained, using shaping procedures, to peck through a layer of white tissue paper and retrieve a mealworm reward concealed in a well, after which, they were tested with a battery of psychometric tests (including those detailed in this study) from 10 days old, with equal exposure in a fixed order to all tasks.

Subjects were presented with two discrimination tasks (2014: spatial location; 2015: colour) involving an acquisition learning phase and a reversal learning phase. Each task required subjects to discriminate between wells whose contents were concealed. Rewarded wells contained a single medium-sized (approx. 2 cm) mealworm, while unrewarded wells remained empty.

In 2014, subjects were presented with a board containing 20 covered wells arranged in two sets of 5 × 2 wells. During the acquisition phase, when the birds were four weeks old, the 10 wells furthest from the entry door were rewarded and the 10 wells closest to the door were unrewarded. During the reversal phase, when the birds were five weeks old, the locations were reversed with wells closest to the door being rewarded and those furthest being unrewarded. The location and order of all wells that the bird pecked at was recorded. Each subject received 10 trials during each phase, meaning that we considered data from 100 well pecks per subject.

In 2015, five-week-old subjects were presented with a refined version of the test apparatus, with pairs of wells presented sequentially. Each well was surrounded by either a blue or green border. Blue-bordered wells were rewarded during the acquisition phase and green wells were rewarded in the reversal phase. The spatial location of each well was pseudo-randomized, with no more than three wells being in the same location in every five pairs that were presented. Subjects were presented with five sets of 10 pairs of wells during each phase, meaning that we considered data from 50 binary choices for each bird in each of the acquisition and reversal tasks.

In both years, we placed a single, uncovered, live mealworm in the middle of the test apparatus to centre the test subject and induce them to interact with the task. Birds entered the testing chamber voluntarily and alone from a communal holding area. On completion of the task, they exited to a second holding area. This ensured that each bird entered the test area once and only once per session.

### (c) Measuring cognitive performance

We measured performance in both the acquisition and reversal tasks as the improvement in the proportion of correct choices made over the testing period. In both years, we subtracted the proportion of correct choices made in their first 15 choices from the proportion of correct choices made in their last 15 choices, only including individuals who completed all choices per trial (some ceased participating before this). We had data for improvements in both acquisition and reversal performance for 80 birds in 2014 and 173 different birds in 2015.

We confirmed that there was learning of the task at the level of the population. In 2014, birds improved by 11% during the spatial acquisition task (one-sample *t*-test: H_0_: *x* = 0, *t*_79_ = 4.79, *p* < 0.0001) and 18% during the reversal task (*t*_131_ = 8.39, *p* < 0.0001). In 2015, birds improved by 24% during the colour acquisition task (*t*_176_ = 15.90, *p* < 0.0001) and 25% during the reversal task (*t*_178_ = 18.54, *p* < 0.0001). Although birds demonstrated a clear improvement in their performances between the start and end of each task, a few individuals exhibited no errors after 50 trials. Therefore, we cannot describe the birds as having learned the affordances of the task, but rather we can only refer to their learning progress over the standardized number of trials. For conciseness, we refer to this as an individual's acquisition or reversal learning speed.

### (d) Assays of early-life exploratory behaviour

In 2015, we conducted a series of behavioural assays on birds aged 31 days, in which we recorded their (i) exploration of a novel environment (over two 1 min periods), (ii) latency to approach a novel object, and (iii) latency to approach an unknown conspecific. These assays followed procedures described in [43], (presented in our electronic supplementary material), which reported high repeatability within individuals (*r* = 0.70; *F*_20,42_ = 2.37, *p* = 0.028). As in [43], we extracted a single principal component (PC) score, with eigenvalue greater than 1. For birds tested in this paper, this explained 54% of the variance in the measures and had positive loading towards activity (Novel environment 1: 0.86; Novel environment 2: 0.90) and negative loading towards time taken to move towards a novel object (−0.58) and time taken to reach a conspecific (−0.53). Therefore, an individual with a high PC score tended to be active in a novel environment and quick to approach both novel objects and conspecifics. Such an individual could be described as bold and exploratory. Conversely, an individual with a low PC score tended to be less active in a novel environment and slow to approach a novel object and their conspecific. Such an individual could be described as inactive, shy and non-exploratory. We used a generalized LM to test whether an individual's probability of survival to four months old (a binary variable with a logit link) was predicted by their sex, mass and exploratory score.

### (e) Release into the wild

In both years, when birds were 10 weeks old, they were mixed together and placed into a large release pen that measured approximately 3000 m^2^ and contained woodland, open areas of grass and dense patches of understory. The pen was surrounded by wire and electric fences to exclude terrestrial predators, but was unroofed, so that birds could disperse from and return to the pen at will and were exposed to the threat of avian predation in the pen. The pen contained water and food ad libitum. No predator control or game shooting occurred on the farm. Away from the release pen we placed a further 36 feeders that dispensed supplementary wheat.

### (f) Measuring fate and movement in the field

In 2014, we monitored feeders with motion-activated cameras. From the photos, we identified birds from their individually numbered tags and thus determined if a bird was alive. The last day that a bird was recorded by the camera was deemed to be their day of death. This measure is imperfect, because the birds could simply have left the study site. However, we believe that dispersal was low for two reasons. First, we radio-tagged a sample of 30 birds and only detected one of these off the site during the four months covered by this study. This matches previous work in which the vast majority of birds stay close to their release pen (95% of locations less than 1.6 km of the release pen [44]). Second, over the following 18 months we engaged in detailed field observations, trapping programmes and additional automated camera monitoring of the site at new locations for other unrelated aspects of a broader study, and during this time we did not detect any birds that we deemed to be dead, confirming that we had not simply failed to detect them.

Despite our confidence that our assumption of death was accurate, we subsequently used a set of more rigorous criteria. In 2015, we made a concerted effort to recover dead birds. This combined radio-tagging birds and regular and detailed searching in the study area. We only included birds as dead if we had found their carcasses or identifiable bits of them within 60 days of their release into the wild. We only included birds as alive if we detected them at feeders (using radio-frequency identification (RFID) tags and readers set at the feeder sites) or observed them directly within 60 days of their release into the wild. Any bird that was not detected and whose fate was ambiguous were excluded from analysis.

We recorded how many different feeders outside the release wood a pheasant visited in the seven months following their release in 2014 and used this as an indicator of their ranging behaviour. Feeders can only be visited by live birds, and the release of birds from a single point followed by their subsequent dispersal makes corrections for time of death difficult. Therefore, we only considered birds that had survived for one season post-release and were known to be alive in March 2015. We considered that a bird had visited a feeder if it was detected there on at least five occasions (to exclude potentially unreliable identifications from the photos). We tested whether an individual's acquisition and/or reversal learning speed predicted the number of feeders they visited post-release. Failures in some of our RFID readers and tags following the release of birds in 2015 meant that we could not obtain reliable movement behaviour for that cohort.

### (g) Determining a meaningful survival threshold

We determined the threshold for survival at 60 days (see electronic supplementary material for our rationale). The carcasses we recovered were all predated or scavenged, and field signs indicated that mammalian predators, probably foxes, were the most common. Because the carcasses had been at least partially eaten, it was not possible to reliably autopsy them, so we cannot be certain that dead birds had not died of starvation or disease and were then scavenged, and cannot determine whether birds in poor nutritional condition were more susceptible to predation.

### (h) Statistical analysis

Our measure of year accounted for differences in weather and other ecological conditions between the years, and also served to account for the differences in the cues used in the cognitive tests (spatial 2014; colour 2015). We constructed a generalized linear model (GLM) with Type III sum of squares considering likelihood ratios, with survival at four months old as our binary response variable with a logit link. To improve interpretation of the model effects, we mean-centred continuous variables (mass at release, improvement in acquisition performance, improvement in reversal performance), but we present the uncentred data in figures. The full model included all five main effects (year, sex, mass at release (g), acquisition learning speed, reversal learning speed) as well as all of the two-way interactions. We present the full model in electronic supplementary material, table S1. We then constructed a minimal model, which initially included all main effects and two-way interactions from which terms were dropped until no improvement was made in model fit indicated by declining AIC values. In 2014, we included 80 birds with known cognitive performances in both acquisition and reversal tasks and imputed fates. In 2015, we included a further 74 birds with known performances in both task types and known fates. No birds appeared in both years. Because we used a different cue set in each year and this may have unspecified interactions with differing environmental conditions in each year, we also tested whether the overall patterns seen across years were present in each year separately by running the minimal model derived from the complete dataset, excluding terms which involved ‘year’. We report these models in the electronic supplementary material.

We used a GLM to ask whether an individual's reversal learning speed was predicted by their acquisition learning speed. We included year as a factor to control for the overall differences in improvements between the two different test paradigms.

Because we only measured post-release ranging behaviour in 2014/15 (see above) and only measured pre-release exploratory behaviour in the 2015 chicks, we had to relate these movement behaviours to cognitive abilities independently for each year in separate analyses. We had records for 41 birds still alive in March 2015 for whom we had measures of cognitive performance and for whom we could investigate their post-release movement behaviour. In 2015, we had exploratory movement assays and cognitive performance measures for 132 birds; for some of these we did not have confirmed fates. Variation in sample sizes between tests are due to some individuals failing to meet inclusion criteria in one task. All analyses were conducted in SPSS v23.

### (i) Ethical note

Work was conducted under UK Home Office licence PPL 30/3204. Birds were habituated to human observation from 1 day old. Shaping procedures, using mealworm rewards, were adopted to habituate subjects to the testing arena. These procedures were considered to alleviate stress and encourage subjects' voluntary participation during testing. Birds could therefore choose whether or not to participate in tasks. There were no enforced aversive stimuli. To encourage participation in the tests, birds were removed from their normal food supply (but not water) for up to 2 h before testing while in the holding section. Birds that failed to engage with the task in less than 2 min were permitted to pass into the recovery area and their lack of participation recorded. Birds were reared at a lower density than that recommended by the UK Department for Environment, Food and Rural Affairs (Defra)'s code of practice, thus probably reducing stress and competition between chicks.

## 3. Results

**Figure 1.**
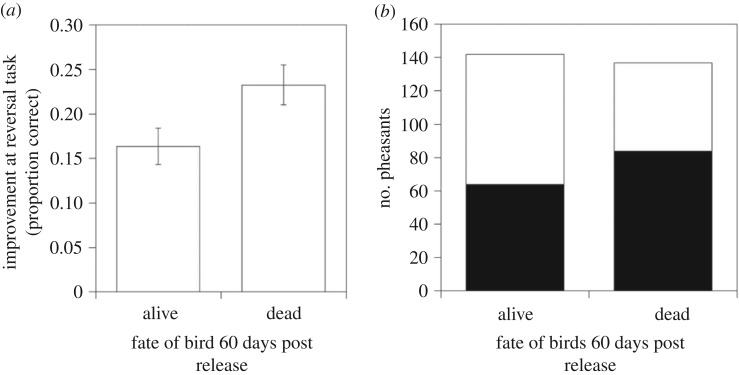
The: (*a*) mean cognitive performance (improvement at a reversal task) when tested at 4–5 weeks old; and (*b*) sex (females = white, males = black); of 154 pheasants that were classed as either alive (*N* = 76) or dead (*N* = 78) 60 days after having been released into the wild. Error bars indicate ±1 s.e.

**Figure 2.**
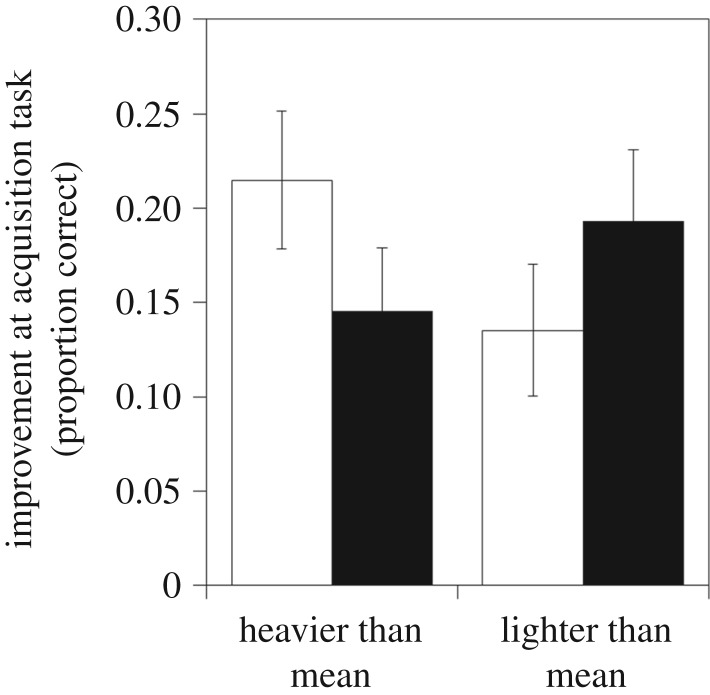
The mean improvement in an acquisition task administered at 4–5 weeks by 154 pheasants that were either heavier or lighter than the population mean when released into the wild at 10 weeks and were classified as either alive (white bars) or dead (black bars) at 60 days after being released into the wild. Error bars indicate ±1 s.e.

Individual pheasants that were slow at learning to reverse a previously learned association when young were more likely to be alive after four months as indicated by both the full and minimal models (figure 1*a* and table 1; electronic supplementary material, table S1). Surviving pheasants had been approximately 30% slower to reverse compared to pheasants that died. This effect was consistent across both years (Reversal Speed*Year: Likelihood Ratio *χ*^2^ = 0.02, *p* = 0.88). For heavy birds, those that were fast learning to acquire an association were more likely to be alive at four months, but for light birds, it was those that were slower to learn to acquire the association that were alive after four months (Body mass*Acquisition Speed: Likelihood Ratio *χ*^2^ = 5.59, *p* = 0.018, figure 2). When we analysed each year separately, despite *β* values for the effects of reversal speed and those of the interaction between mass and acquisition speed being similar across years, the effects were only significant for reversal speed in 2014 (electronic supplementary material, table S2).

**Table 1.** Model output from GLM exploring predictors of a pheasant's probability of surviving at least 60 days after release. This is the minimum model based on a stepwise deletion of predictors based on AIC values. See electronic supplementary material, table S1 for the full model. Reference categories for *β* values: year = 2015; sex = female.

	*β*	likelihood ratio *χ*^2^	d.f.	*p*
intercept	1.09	0.34	1	0.56
year	−0.20	2.36	1	0.12
sex	−2.68	12.49	1	*<0.001*
mass	0.01	5.46	1	*0.019*
reversal speed	−2.26	6.87	1	*0.009*
mass * acquisition speed	0.021	5.59	1	*0.018*
year * sex	1.58	2.18	1	0.14
year * mass	−0.008	2.24	1	0.13

Males were disproportionately likely to die by four months compared with females as indicated by both the full and minimal models (figure 1*b* and table 1; electronic supplementary material, table S1). Birds that were heavy when released were more likely to be alive after four months (table 1; electronic supplementary material, table S1).

An individual's reversal learning speed did not significantly predict their acquisition learning speed although the slope relating the two measures was positive (*β* = 0.173, 95% CI = 0.008–0.337) (GLM: *F*_1,253_ = 2.41, *p* = 0.12). The extent to which birds learned the tasks differed between the years (GLM: *F*_1,253_ = 5.04, *p* = 0.023), probably because of the different test paradigms used, but there was no interaction between the two tests and year (*F*_1,253_ = 1.54, *p* = 0.24).

In 2014, individuals who had high acquisition learning speeds were observed at a larger number of feeders outside of their release pen (*r*_41_ = 0.362, *p* = 0.020, electronic supplementary material, figure S2). An individual's reversal learning speed was not related to the number of feeders that they visited (*r*_69_ = 0.17, *p* = 0.15). In 2015, individuals with fast reversing speeds were also those scoring highly on the component that indicated high exploration in early life (*r*_132_ = 0.209, *p* = 0.016, electronic supplementary material, figure S1). However, an individual's acquisition learning speed did not relate to assays of their early life exploratory behaviour (*r*_131_ = 0.10, *p* = 0.28). An individual's exploratory score did not predict their probability of survival (electronic supplementary material, table S3).

## 4. Discussion

Early life performance in two cognitive tasks predicted the mortality of released pheasants. These effects were detected when we considered the combined performances from tasks based on two types of cues (spatial and colour), conducted over 2 years in a large sample of 154 birds and controlled for other factors likely to influence survival, namely sex and body mass. Pheasants that exhibited slow reversal learning speeds were more likely to survive when released into the wild. Pheasants with fast acquisition learning speeds and that were larger than average were more likely to survive, whereas those fast acquirers that were smaller than average were less likely to survive than slow acquirers.

The use of different cue sets in different years risks a potential confounding interaction between year and task. Perhaps something about the environment in 2014 was especially likely to interact with performance in a spatial acquisition and reversal, whereas in 2015 a different factor, absent or less influential in 2014, interacted with colour abilities, but we cannot conceive of what these specific factors may be. Ideally, the two different sets of cues should have been used in each of the 2 years across all birds in order to, first, allow us to confirm the repeatability of an individual's performance across contexts (Cauchoix *et al*. [45]), and, second, exclude the possible interactions between the environment and cue sets. However, the logistics of conducting our tests in those years precluded this more robust experimental design. Therefore, it is reassuring to see that the *β* values for the effects of reversal speed and the interaction between mass and acquisition speed remained similar across years (electronic supplementary material, table S2). This suggests that the effects of cognitive performance on survival are somewhat weak and therefore we believe that detecting fitness consequences of cognitive abilities may require subtle, long-term and large-scale studies.

The positive relationship between an individual's acquisition learning speed and their likelihood of surviving is perhaps not surprising. The ability to rapidly form an association has previously been demonstrated to bring fitness benefits in the form of improved growth rates for grasshoppers and offspring production for parasitoid wasps [20,21]. We are not certain why this relationship was stronger for pheasants that were heavier at release compared with light pheasants. One explanation is that a learned novel resource could be most effectively exploited by more dominant individuals, if larger birds are more dominant, or larger socially dominant birds may have more opportunities to access novel resources and hence learn about them. Alternatively, the effect may arise independently of a link between learning speed and survival. Across species, larger individuals may have been better fed and/or exhibit higher metabolic rates, and hence be more motivated to learn a food-rewarded task (bumblebees (*Bombus impatiens*) and zebra finches (*Taeniopygia guttata*): [46,47]). Overall, and after accounting for sex, larger pheasants were more likely to survive. This matches findings in previous studies of wild pheasant populations (in the USA) which have suggested that larger pheasants are more likely to survive because they have greater nutrient reserves, are more mobile and hence can move more easily to preferred locations, or they are simply harder for predators to kill [39].

The negative relationship between reversal learning speed and an individual's survival is unexpected. The performance of red jungle fowl in a reversal task (but not the corresponding acquisition) was moderately heritable (Sorato *et al*. [48]). An alternative assay of inhibitory control, performance in a detour-reaching task, was positively related to a fitness proxy (male song repertoire) in song sparrows [10] although performance in a reversal task by the same birds was unrelated to song. A negative relationship between cognitive performance and fitness has only been reported in a single study such that male song sparrows that were fast at spatial learning also had smaller song repertoires [14]. A negative relationship implies that selection could act against a particular facet of cognitive performance. We suggest three explanations as to why such a negative relationship may occur.

First, there may be physiological, neurological or psychological costs leading to a trade off with another cognitive ability. Exaggeration of cognitive abilities may be constrained by energetic, neurological or psychological processes. Investment in neural (typically brain) tissue, which is considered to correspond to cognitive performance (e.g. [49,50], but see also [51]), is expensive, and individuals selected for larger brain size suffer a series of corresponding fitness costs and trade-offs against the development of other organs [43]. Operating neural tissue is energetically costly with increased computational load incurring increasing costs [52]. If increased cognitive performance demands greater neural processing, then such energetic costs must be faced. Given a limited quantity of neural tissue, we might expect trade-offs between different types of cognitive mechanisms. For example, two common cognitive mechanisms (acquisition and reversal learning) involve different neuronal mechanisms and brain regions [53–55]. Investment in one area reduces resources and space available to construct, maintain and operate the other region. We found no evidence for such a negative relationship between acquisition and reversal learning speeds. Instead, the slope of our non-significant relationship was positive. Consequently, we are sceptical that the importance of slow reversal speed is simply the inevitable consequence of enhanced acquisition learning.

A second reason why individuals with low reversal speeds may be more likely to survive is that their cognitive performance corresponds to a broader personality type governing a suite of behaviours that influence mortality in concert with one another. Links between an individual's cognitive performance and their personality have been reported [30,31] as have links between personality and fitness outcomes [56]. For pheasants, individuals that were shy or slower to explore novel situations as juveniles were more likely to survive a hunting season overall, perhaps because bold males were likely to be shot earlier in the season, although males that died of disease or predation were relatively bold or fast as juveniles, while females dying of disease or predation were relatively shy or slow [57]. Pheasants in the current study that showed low levels of exploratory behaviour in artificial testing chambers when young were also those demonstrating slow reversal learning speeds when young. A positive relationship (slow explorers are slow reversers) between extent of exploration and reversal speed has been reported in great tits (*Parus major*) and black-capped chickadees [58–60]. However, in another population of chickadees [27] and Florida scrub jays [25], individuals described as reactive, timid, less explorative or less aggressive were faster learners at reversal tasks than bolder individuals. An exploratory personality type may drive birds to leave the safety of the release pen, where a fence protected them from terrestrial, but not aerial, predators and venture into new areas where predation risk was higher or food supplies were lower. If so, selection may act on the exploratory behaviour with indirect pleiotropic effects on speed of reversal. We believe this was not the case with our pheasants because among pheasants that survived the season, we did not find that performance in the reversal tasks related to the number of feeders a bird visited. Furthermore, we did not find a direct relationship between an assay of an individual's exploratory behaviour when young and their probability of survival. This suggests that the roles of exploratory personality type and cognitive performance in the reversal task act somewhat independently of one another.

Finally, perhaps poor flexibility or an inability to inhibit learned positive associations is itself adaptive, at least in some circumstances. We are not aware of tests which demonstrate that individuals may benefit from cognitive abilities that are not the most extreme of variants. Studying and discussing this area can be complicated by the use of language in which there is commonly a presumption that exaggeration of cognitive abilities is inherently beneficial. Individuals that learn slowly, exhibit restricted memory span or exert low levels of executive control are frequently described as having ‘poor’ cognitive performance (e.g. [50]). Such subjective labelling may serve to reinforce the assumption that selection favours particular directions of cognitive exaggeration and hence inhibits researchers from searching for, or publishing negative relationships. Situations where poor performance in a cognitive domain corresponds to fitness benefits is seen in prairie voles (*Microtus ochrogaster*) where males with neural and genetic correlates of impaired spatial memory get lost, wander and hence meet more females, resulting in increased reproductive success [61], and in great tits where females with lower problem-solving performance were less likely to abandon nests, even though they laid smaller clutches [62]. Pheasants in the UK live in landscapes that are managed to enhance their survival (at least up to the start of the hunting season). For example, at our site we provided feeders filled with grain that pheasants could access ad libitum. In such managed landscapes where food resources were predictable, stable and relatively easily available, it may be beneficial to learn a single strategy, such as targeting a particular feeder location, and then inflexibly stick to it rather than continually switching to alternative opportunities. This would reduce the area that they range over and consequently reduce the number of different predators they may encounter. Additionally, spending a long time in a restricted area may allow pheasants to develop a detailed knowledge of that local area including refuges. Therefore, the costs of behavioural flexibility that we detected may be unusual to this particular context.

Our findings that apparently poor cognitive performance correlates with fitness benefits may complement observations from selection experiments in which extremely ‘good’ or poor performers are artificially selected and bred together, and the behaviour and fate of resulting generations of offspring are recorded. This approach reveals a negative relationship between cognitive performance and fitness, opposite to that of the correlative approach used by us and others. Fruit flies (*Drosophila melanogaster*) bred for improved learning ability had shorter lifespans and lower competitive abilities as larvae [63–65]. Fruit flies bred for improved long-term memories were more susceptible to stress in the form of desiccation [64]. Male worms (*Caenorhabditis remanei*) selected for improved olfactory learning were less active and had lower survival, but they did sire more offspring [66]. Selection for large brains in guppies, which led to large-brained females outperforming small-brained females in a numerical learning task, also tended to produce fewer offspring [43] although they survived better when housed with predators [67]. Such selection studies reveal that ‘better’ cognitive abilities may incur fitness costs, through a mediating factor such as energetic costs and developmental trade-offs, and therefore continued exaggeration of cognitive abilities may be constrained. We did not measure differential energetic costs paid by fast and slow learners. It is possible that pheasants with fast reversal learning speeds bore higher energetic demands to support their neural architecture which in turn either forced them to forage for longer in exposed locations or otherwise was detrimental to their health. We found no evidence that on release pheasants with either fast acquisition or reversal learning speeds had lighter body masses; neither did we find any dead pheasants that had starved. Consequently, we do not believe that the costs of exhibiting particular speeds of acquisition or reversal are directly due to energetic demands.

The correlation we find between an individual's early life performance in cognitive tasks and their later survival suggests two further mechanisms by which cognitive performance may be constrained. First, the benefits of enhanced cognitive performance may be dependent on other attributes. In the case of pheasants, for heavy birds, selection may favour enhanced acquisition speed, whereas for lighter birds, selection instead favours reduced acquisition speed. Consequently, selection for particular exaggerated cognitive performance may be stabilized by selection on other non-cognitive traits, altering the adaptive value of the cognitive ability. Second, exaggerated cognitive performance, which might be expected to bring benefits in some contexts, may actually permit or even encourage individuals to engage in behaviours that are risky, either through pleiotropy or correlated behavioural syndromes. In the case of pheasants, we found that individuals with fast reversal learning speeds were also more exploratory in a range of contexts when young. Such behavioural flexibility and exploratory behaviour may be beneficial in some contexts, but in managed farmland where the pheasants were released it encourages them to leave areas with consistent food supplies and protection from predators and venture into new areas where they risk starvation and predation.

The correlations we report suggest that exaggerated cognitive performance may lead to maladaptive, costly behavioural outcomes, at least under some circumstances, and this should cause us to examine the relationships between cognition and fitness more carefully. Such relationships may be weak and may only manifest under particular environmental conditions. A negative relationship between an individual's cognitive ability and their fitness helps understand why relatively energetically cheap exaggeration of cognitive abilities may not be beneficial. It might explain why individuals and species differ in the expression of their cognitive abilities and why we do not commonly see instantaneous learning, perfect memory or immediate and flexible executive control.

## Supplementary Material

Supporting Methods and Results

## Data accessibility

Data are available in DRYAD: Data from: The quick are the dead: pheasants that are slow to reverse a learned association survive for longer in the wild. http://dx.doi.org/10.5061/dryad.9bn4443.
