# Supplementary material for: The quick are the dead: pheasants that are slow to reverse a learned association survive for longer in the wild
Source: Philos Trans R Soc Lond B Biol Sci. 2018 Aug 13;373(1756):20170297. doi: 10.1098/rstb.2017.0297 (PMC6107567; doi:10.1098/rstb.2017.0297)
Supplement: Supporting Methods and Results [file rstb20170297supp1.docx]

ESM for:

**The quick are the dead: Pheasants that are slow to reverse a learned association survive for longer in the wild**

Joah R. Madden*, Ellis J.G. Langley, Mark A. Whiteside, Christine E. Beardsworth, Jayden O. van Horik

**Husbandry and sources of pheasants**

In 2014 we bought the chicks form a commercial game breeder who maintained a large and well-mixed breeding stock. In 2015, we brought 140 chicks from the same dealer and reared the rest ourselves from a well-mixed population of adults that we had caught ourselves. These adults contained some individuals who had been reared and released by us in 2014 and other adults that were not involved in our study in 2014. All pheasants were individually marked using numbered wing tags, fed commercial pheasant feed supplemented with wild bird seed (~5%) and supplied with water *ad libertum.* Birds were housed in 2m x 2m heated huts for the first 2 weeks of life. Chicks had access to an unheated but covered outdoor runs of 1m x 4m for the following week, and had access to 4m x 12m uncovered outdoor runs for the final seven weeks of rearing. Connected to the rearing enclosure, and separated with a sliding door, was a 75x75cm testing arena.

**Tests of exploratory behaviours**

*Response to a novel environment*

A bird was caught from its rearing pen using a hand net and immediately placed into a plastic crate measuring 0.95 × 0.65 m and 0.27 m high. The crate floor was covered with a green Styrofoam board marked out in 20 cm squares and the crate walls were covered with the Styrofoam sheets ensuring that the bird was in visual but not auditory isolation from others. The bird was placed into the central square and left for 1 min. We measured the individual's response to this novel environment by determining its activity level, directly observing the bird from above while positioned behind a screen, and counting the squares entered during a 1 min focal follow. A new square was entered when the outside toe of the right foot was placed in a square separate from its previous position. The same square that was left and subsequently re-entered was counted twice.

*Response to a novel object*

Immediately after Test 1 (Response to a novel environment) was completed, we placed a novel object (a blue beaker measuring 8 cm diameter × 17 cm tall) three squares away from the bird. The beaker was selected because it was of a colour and shape not encountered in the rearing environment. We then timed how long the bird took to touch the beaker with any part of its body (precision = 1 s). If birds took longer than 3 min, we recorded a time of 180 s and ended the test.

*Response to novel social stimuli*

Immediately after Test 2 (Response to a novel object) was completed, we removed half of one of the sheets on the wall of the crate to expose a neighbouring crate in which we placed five other mixed-sex, 4-week-old pheasants that were unknown to the test bird. We moved the test bird to the square furthest from the exposed crate and then timed how long it took the test bird to place the outside toe of its right foot into the square closest to the crate. If birds took longer than 3 min, we recorded a time of 180 s and ended the test.

**Determining a meaningful survival threshold**

We determined the threshold for survival at 60 days (see ESM for our rationale). for two reasons. First, survival rates of released pheasants can be extremely low, being consistently around only ~20% after 60 days post-release across a range of habitats where predator control is low [1-3], with UK predators including foxes and raptors [4]. Our putative survival curve based on observations in 2014 was greater than this, with 102/197 (52%) released birds known to be alive at 60 days after their release despite our practicing no predator control on the site. The steep decline in survival appears to slow around 60 days with 61/197 (31%) still alive 200 days after release. Secondly, this date corresponded to the week that pheasant shooting is permitted in the UK, and therefore offers a practical argument. Consequently, mortality after this point could have been due to anthropogenic rather than natural causes. All birds considered in this analysis died of natural causes and were not shot.

**ESM References**

1. Robertson, P. A. (1988). Survival of released pheasants, *Phasianus colchicus*, in Ireland. *Journal of Zoology*, *214*(4), 683-695.
2. Hessler, E., Tester, J. R., Siniff, D. B., & Nelson, M. M. (1970). A biotelemetery study of survival of pen-reared pheasants released in selected habitats. *The Journal of Wildlife Management*, 34(2), 267-274.
3. Krauss, G. D., Graves, H. B., & Zervanos, S. M. (1987). Survival of wild and game-farm cock pheasants released in Pennsylvania. *The Journal of Wildlife Management*, 555-559.
4. Sage, R. B., Turner, C. V., Woodburn, M. I., Hoodless, A. N., Draycott, R. A., & Sotherton, N. W. (2018). Predation of released pheasants *Phasianus colchicus* on lowland farmland in the UK and the effect of predator control. *European Journal of Wildlife Research*, *64*(2), 14.

**ESM Tables**

|  | **β** | **Likelihood Ratio χ^2^** | **DF** | **P** |
| --- | --- | --- | --- | --- |
| Intercept | 0.41 | 0.41 | 1 | 0.52 |
| Year | 2.39 | 2.39 | 1 | 0.12 |
| Sex | 11.07 | 11.07 | 1 | **0.001** |
| Mass | 3.48 | 3.48 | 1 | 0.062 |
| Acquisition speed | 0.12 | 0.12 | 1 | 0.73 |
| Reversal speed | 4.42 | 4.42 | 1 | **0.036** |
| Year * Sex | 1.54 | 1.54 | 1 | 0.22 |
| Year * Mass | 1.87 | 1.86 | 1 | 0.17 |
| Year * Acquisition speed | 1.30 | 1.30 | 1 | 0.25 |
| Year * Reversal speed | 0.024 | 0.02 | 1 | 0.88 |
| Sex * Mass | 0.042 | 0.04 | 1 | 0.84 |
| Sex * Acquisition speed | 0.35 | 0.35 | 1 | 0.55 |
| Sex * Reversal speed | 0.24 | 0.24 | 1 | 0.62 |
| Mass * Acquisition speed | 0.73 | 0.73 | 1 | 0.39 |
| Mass * Reversal speed | 1.00 | 1.00 | 1 | 0.32 |
| Acquisition speed * Reversal speed | 0.06 | 0.06 | 1 | 0.80 |

**Table 1** Model output from GLM exploring predictors of a pheasant’s probability of surviving at least 60 days after release. The full model with all main effects and 2 way interactions included. Reference categories for β values: year = 2015; sex = female.

A) 2014

|  | β | Likelihood Ratio χ^2^ | df | P |
| --- | --- | --- | --- | --- |
| Intercept | -0.93 | 2.82 | 1 | 0.093 |
| Sex | 1.14 | 2.50 | 1 | 0.11 |
| Mass | -0.002 | 0.34 | 1 | 0.56 |
| Reversal speed | 2.36 | 4.73 | 1 | 0.030 |
| Mass * Acquisition speed | -0.025 | 3.32 | 1 | 0.068 |

B) 2015

|  | β | Likelihood Ratio χ^2^ | df | P |
| --- | --- | --- | --- | --- |
| Intercept | -1.07 | 5.10 | 1 | 0.024 |
| Sex | 2.59 | 8.81 | 1 | 0.003 |
| Mass | -0.01 | 4.53 | 1 | 0.033 |
| Reversal speed | 2.00 | 1.52 | 1 | 0.22 |
| Mass * Acquisition speed | -0.018 | 1.96 | 1 | 0.16 |

**Table 2** Outputs from the minimal model (excluding terms involving ‘year’) exploring predictors of a pheasant’s probability of surviving at least 60 days after release based on data from only A) 2014 and B) 2015. Reference categories for β values: sex = female.

|  | **β** | **Likelihood Ratio χ^2^** | **DF** | **P** |
| --- | --- | --- | --- | --- |
| Intercept | 5.32 | 0.92 | 1 | 0.34 |
| Sex | 2.29 | 7.13 | 1 | 0.008 |
| Mass | -0.008 | 2.73 | 1 | 0.098 |
| Exploration | -0.32 | 0.87 | 1 | 0.35 |

**Table 3** Model output from GLM exploring how personality score predicts a pheasant’s probability of surviving at least 60 days after release in 2015. Reference categories for β values: sex = female.

**ESM Figures**


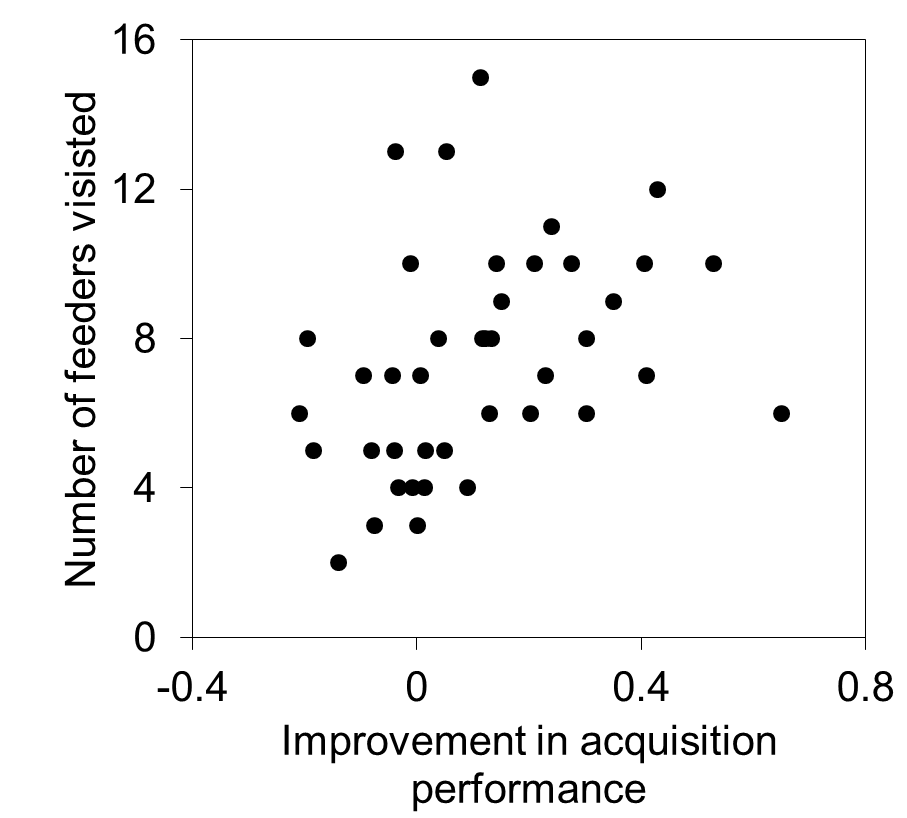


**Figure 1.** The relationship between the improvement in an individual’s performance on an acquisition task when 4-5 weeks old and the numbers of feeders that they visited between their release into the wild at 10 weeks in August 2014 and the end of our monitoring in March 2015 for 41 pheasants.


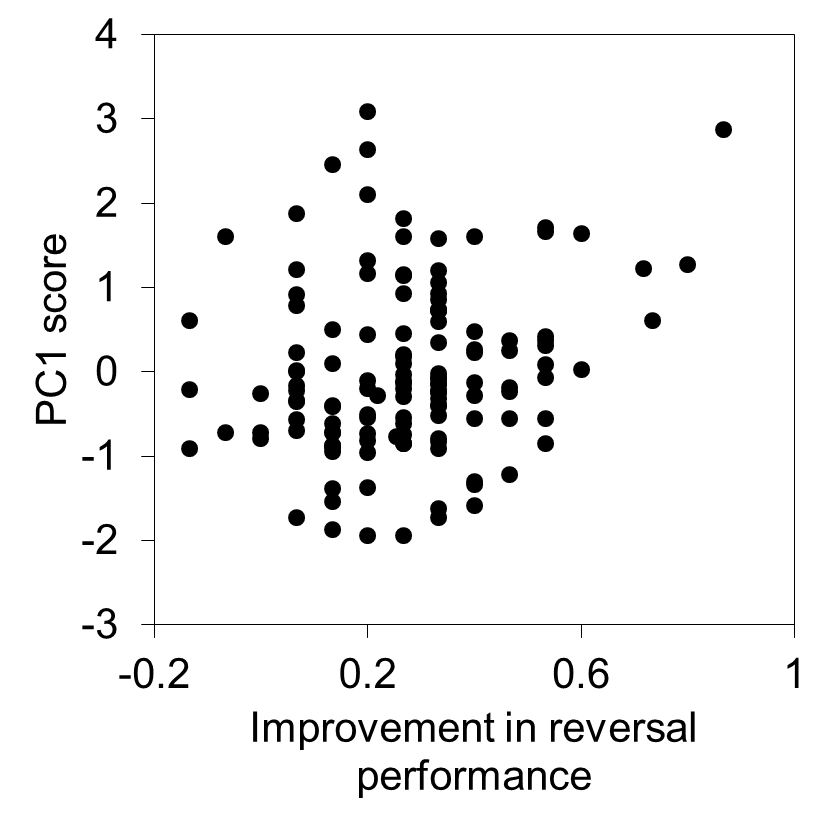


**Figure 2.** The relationship between the improvement in an individual’s performance on a reversal task when 4-5 weeks old and the first PC summarising their behaviour in a set of personality tests for 131 birds assayed in 2015. A higher, positive PC score indicates a bolder and more exploratory bird and a lower, negative score indicates a shyer, less exploratory bird.
